# Enrichment of Zinc and Iron Micronutrients in Lentil (*Lens culinaris* Medik.) through Biofortification

**DOI:** 10.3390/molecules26247671

**Published:** 2021-12-18

**Authors:** Salwinder Singh Dhaliwal, Vivek Sharma, Arvind Kumar Shukla, Janpriya Kaur, Vibha Verma, Prabhjot Singh, Harkirat Singh, Shams H. Abdel-Hafez, Samy Sayed, Ahmed Gaber, Reham Ali, Akbar Hossain

**Affiliations:** 1 Department of Soil Science, Punjab Agricultural University, Ludhiana 141004, India; ssdhaliwal@pau.edu (S.S.D.); sharmavivek@pau.edu (V.S.); janpriyakaur89@pau.edu (J.K.); vermavibha@pau.edu (V.V.); prabh@pau.edu (P.S.); dhaliwalss764@gmail.com (H.S.); 2 Indian Institute of Soil Science (IISS), Bhopal 462038, India; arvindshukla2k3@yahoo.co.in; 3 Department of Chemistry, College of Science, Taif University, Taif 21944, Saudi Arabia; s.abdelhafez@tu.edu.sa; 4 Department of Science and Technology, University College-Ranyah, Taif University, Taif 21944, Saudi Arabia; s.sayed@tu.edu.sa; 5 Department of Biology, College of Science, Taif University, Taif 21944, Saudi Arabia; 6 Department of Chemistry, College of Science, Qassim University, Buraidah 51452, Saudi Arabia; re.ali@qu.edu.sa; 7 Chemistry Department, Science College, Suez University, Suez 43518, Egypt; 8 Department of Agronomy, Bangladesh Wheat and Maize Research Institute, Dinajpur 5200, Bangladesh

**Keywords:** lentil, micronutrient content, foliar application, efficiency indices, economic feasibility

## Abstract

Biofortification of pulse crops with Zn and Fe is a viable approach to combat their widespread deficiencies in humans. Lentil (*Lens culinaris* Medik.) is a widely consumed edible crop possessing a high level of Zn and Fe micronutrients. Thus, the present study was conducted to examine the influence of foliar application of Zn and Fe on productivity, concentration, uptake and the economics of lentil cultivation (LL 931). For this, different treatment combinations of ZnSO_4_·7H_2_O (0.5%) and FeSO_4_·7H_2_O (0.5%), along with the recommended dose of fertilizer (RDF), were applied to the lentil. The results of study reported that the combined foliar application of ZnSO_4_·7H_2_O (0.5%) + FeSO_4_·7H_2_O (0.5%) at pre-flowering (S1) and pod formation (S2) stages was most effective in enhancing grain and straw yield, Zn and Fe concentration, and uptake. However, the outcome of this treatment was statistically on par with the results obtained under the treatment ZnSO_4_·7H_2_O (0.5%) + FeSO_4_·7H_2_O (0.5%) at S1 stage. A single spray of ZnSO_4_·7H_2_O (0.5%) + FeSO_4_·7H_2_O (0.5%) at S1 stage enhanced the grain and straw yield up to 39.6% and 51.8%, respectively. Similarly, Zn and Fe concentrations showed enhancement in grain (10.9% and 20.4%, respectively) and straw (27.5% and 27.6% respectively) of the lentil. The increase in Zn and Fe uptake by grain was 54.8% and 68.0%, respectively, whereas uptake by straw was 93.6% and 93.7%, respectively. Also the benefit:cost was the highest (1.96) with application of ZnSO_4_·7H_2_O (0.5%) + FeSO_4_·7H_2_O (0.5%) at S1 stage. Conclusively, the combined use of ZnSO_4_·7H_2_O (0.5%) + FeSO_4_·7H_2_O (0.5%) at S1 stage can contribute significantly towards yield, Zn and Fe concentration, as well as uptake and the economic returns of lentil to remediate the Zn and Fe deficiency.

## 1. Introduction

Pulses and legumes belong to the nutritionally rich Fabaceae family and are an important source of plant proteins, having a low glycemic index. Apart from that, they are great reservoirs of vitamins, minerals and complex carbohydrates that are crucial for optimum growth as well as development [1]. Among different food legumes, lentil (*Lens culinaris* Medik.) is a vital grain legume crop grown worldwide, and is also considered as the cheapest source of protein and micronutrients such as Zn and Fe with the concentrations ranging from 44–54 mg·kg^−1^ and 73–90 mg·kg^−1^, respectively [2,3]. However, a wide variation in micronutrient content has been observed among diverse lentil genotypes [4,5]. It is a widely used ingredient in many plant-based diets because of its lower-cooking time, and economically available minerals and good-quality proteins [6,7]. However, the presence of phytate phosphorus in sufficient amount limits the bioavailability of Zn and Fe for absorption in humans [8,9,10]. Moreover, Fe in plants is present as a non-heme in contrast to animal sources, where it is present as heme Fe and thus its bioavailability is relatively lower than animal-based proteins [11].

Intense agricultural activities due to the burgeoning human population have resulted into micronutrient deficiencies in soil. Zinc deficiency is prevalent worldwide, especially in calcareous soils. In India, 51.2 and 19.2% of soils were found to be deficient in Zn and Fe, respectively [12]. Micronutrient deficiencies in soils and crops have led to severe consequences, including reduced yield and low micronutrient concentration in crops, thus resulting in micronutrient malnutrition in humans and animals. Worldwide, Zn and Fe deficiencies have affected one-fifth and one-third of the population, respectively [13]. Zinc deficiency has been observed mainly in developing countries [14]. It is essential for optimum growth and functioning of immune system, enzyme catalyzed biochemical reactions, neurobehavioral development, protein and DNA synthesis [15] and its deficiency results in adverse health conditions such as diarrhea, stunted physical and mental growth, loss of appetite, etc. Iron deficiency results into anemia, which globally has affected 40.0 and 42.0% of pregnant women and children, respectively, and even resulted to 20.0% of maternal deaths [16,17]. Inadequate Fe intake causes the increased mortality of pregnant women and newborns and compromises the immune system [18].

Various approaches such as biofortification, nutrient supplementation, fortification, and diet diversification have been implemented in order to overcome micronutrient malnutrition [19]. However, biofortification is considered as a long-term and efficient method over the others due to the lack of socio-economic infrastructure [20,21,22]. Biofortification also results in better crop productivity and nutritional quality, thus diminishing the micronutrient malnutrition in consumers [23]. Agronomic biofortification refers to the use of mineral fertilizers to improve concentration of nutrients in edible crops and also positively affects the crop yield [24]. The application of fertilizers can be done through soil, seeds and foliar spray; however, foliar application is considered as the best method of increasing the micronutrient level in crops, as nutrients are directed towards the leaves at suitable growth stages. The quick absorption of nutrients promotes nutrient translocations in edible grain parts and avoids nutrient losses in the environment. In addition to this, it promotes plant growth even in less favorable weather conditions.

Several research studies have reported the increase in bioavailability of micronutrients through foliar application. The foliar application of Se at full bloom stage of lentil has been demonstrated to increase the seed Se concentration significantly [25]. Another report has shown the significant positive impact of foliar Zn application on yield and Zn concentration of diverse mungbean genotypes [26]. The application of Zn and Fe sources through foliar spray has proved to be the effective treatment to improve the yield and micronutrient content in rice [27]. Foliar application of Zn reported a significant improvement in plant height, root length, number of nodules, chlorophyll content and seed yield in lentil [28]. Another study reported the positive effect of Zn and Fe foliar application on yield attributes of lentil in the soils of Dylaman, Guilan province, Iran [29]. To the best of our knowledge, no work has reported the effect of the combined application of Zn and Fe on yield, concentration and uptake in lentil in the sandy loam soil of northwestern India. Therefore, the present work was aimed at assessing the influence of foliar application of Zn and Fe on yield, concentration, their uptake, efficiency indices and economic outcomes from lentil cultivation.

## 2. Materials and Methods

### 2.1. Experimental Site and Characteristics

**Figure 1:**
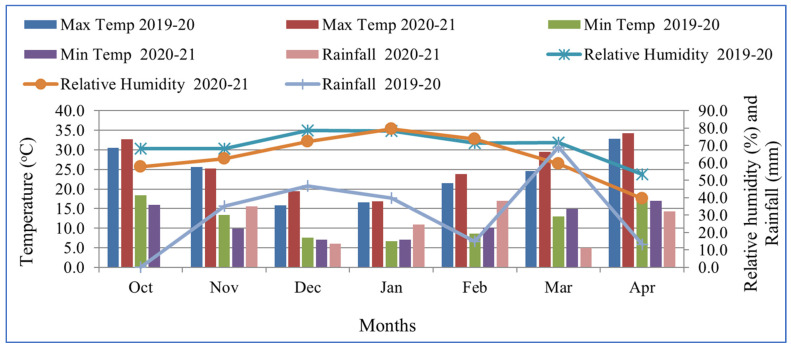
Monthly average maximum and minimum temperature, relative humidity and rainfall of the study area.

The two-year experiment (2019–2020 and 2020–2021) was conducted during *Rabbi* season (November-April) at the experimental farm, Department of Soil Science, Punjab Agricultural University, Ludhiana, Punjab. The research trial was conducted under the bio-fortification mandate of AICRP-MSN project, fully funded by IISS (ICAR), Bhopal, India. The sandy loam soil of this experimental field exhibited a pH of 7.21, electrical conductivity (EC ) 0.34 dS·m^−1^ and organic carbon (OC) 0.31%. The initial level of diethylene triamime penta acetic acid (DTPA) -extractable micronutrients viz. Zn, Cu, Fe and Mn in soil were 1.16, 0.65, 4.86 and 3.91 mg·kg^−1^, respectively. The climate in the region is subtropical, with hot, wet summers and dry winters. The annual rainfall of 400–600 mm was observed with the months of July to September receiving the majority of the rainfall, which is approximately 70% of the total. The total rainfall during the crop season from October to April was 219 and 68.9 mm during 2019–2020 and 2020–2021, respectively. During the growth season of lentil, the average monthly maximum temperature in the study region varied between 15.9 °C and 32.8 °C in 2019–2020 and 16.4 °C and 34.2 °C in 2020–2021, but the minimum temperature varied between 6.7 °C and 18.4 °C in 2019–2020 and 7.1 °C and 17.0 °C in 2020–2021 (Figure 1).

### 2.2. Treatment Details

The study involved different combinations of RDF with ZnSO_4_.7H_2_O (0.5%) and FeSO_4_.7H_2_O (0.5%) i.e., 1.25 kg·ha^−1^ fertilizer applied at pre-flowering (S1) and pod formation (S2) stages i.e., 80 and 100 days after sowing, respectively. For fertilizer application, 250 L·ha^−1^ water was used, thus, 1.25 kg·ha^−1^ fertilizer was applied for each 0.5% fertilizer application. In total, seven treatments were involved along with three replications in a complete randomized block design. The treatment details are as follows (Table 1). The field was plowed twice followed by planking. Nitrogen and P_2_O_5_ with the recommended doses of 12 kg·ha^−1^ and 40 kg·ha^−1^, respectively, were applied as basal doses through urea, diammonium phosphate at the time of sowing. The variety LL 931 was sown in the first week of November, whereas the harvesting was carried out in April (third week). The sowing was performed by the pora method with row to row spacing of 22.5 cm and plot size 3 m × 3.2 m.

### 2.3. Plant Harvesting and Analysis

Plants were harvested manually when they attained physiological maturity followed by the collection of grain and straw samples for analysis (500–550 plants per plot). The samples were air-dried before drying in an oven at 65 °C for 48 h in order to determine the dry weights of plant components. Oven-dried plant samples were further grounded to fine material using a mechanical grinder. A representative grounded straw sample of 1.0 g and grain sample of 0.5 g were digested using a di-acid mixture comprising of HNO_3_ and HClO_4_ acid in ratio 3:1 on an electric hot plate [30]. The micronutrients (Fe, Mn, Zn and Cu) concentration in the digested plant extracts was determined through atomic absorption spectrophotometer (Model AAS 240 FS, Varian, München, Germany). The uptake of micronutrients by lentil grains and straw was calculated by using following equation.

(1) $$Fe\;uptake\;in\;grain\;or\;straw\;\left(g\cdot ha^{-1}\right)=\frac{Yield\;\left(kg\cdot ha^{-1}\right)\times Concentration\;\left(mg\cdot kg^{-1}\right)}{10^{3}}$$

### 2.4. Zn and Fe Use Efficiency Indices

The mobilization efficiency index (*MEI*) was calculated by using the following equation:

(2) $$MEI=\frac{\mathrm{Nutrient}\;\mathrm{concentartion}\;\mathrm{in}\;\mathrm{grain}}{\mathrm{Nutrient}\;\mathrm{concentration}\;\mathrm{in}\;\mathrm{straw}\;}$$

The physiological efficiency of Zn and Fe viz. (PE_Zn_), (PE_Fe_), the apparent recovery efficiency of Zn (ARE-Zn), Fe (ARE-Fe) and the mobilization efficiency index (MEI-Zn), (MEI-Fe) of foliar-applied Zn and Fe were determined with the following equations [31].

(3) $$PE=\frac{Y_{t}-Y_{c}}{NU_{t}-NU_{c}}$$

(4) $$ARE=\frac{NU_{t}-NU_{c}}{Nutrient\;applied\;\left(\mathrm{kg}\cdot {\mathrm{ha}}^{-1}\right)}\times 100$$

where *Y_t_* and *Y_c_* represent the grain yield (kg·ha^−1^) of lentil in fertilized plots and in control, respectively; *NU_t_* and *NU_c_* represent the total nutrient (Zn, Fe) uptake (kg·ha^−1^) of lentil in fertilized plots and in control, respectively.

### 2.5. Economic Analysis

The cost of fertilizer in US dollars (USD) ha^−1^ for various treatments in the experiment was calculated separately, considering the prevailing prices of fertilizers in USD at the time of their use. The price of ZnSO_4_.7H_2_O (0.5%) and FeSO_4_.7H_2_O (0.5%) were 6.67 USD kg^−^^1^ and 8.01 USD·kg^−^^1^, respectively. The price of seed and the selling price were taken as 1.60 USD·kg^−^^1^ and 0.80 USD·kg^−^^1^, respectively, whereas the standard labour cost was 4.94 USD. Gross return (value of additional yield) was calculated based on the MSP (price for minimum support) of lentil by the Indian government during the years of study. Net return (USD·ha^−1^) was obtained by subtracting the fertilizer cost from the gross return as given below.

(5) $$\mathrm{Net}\;\mathrm{Return}\;\left(\mathrm{USD}\cdot {\mathrm{ha}}^{-1}\right)=\mathrm{Gross}\;\mathrm{return}\;\left(\mathrm{USD}\cdot {\;\mathrm{ha}}^{-1}\right)-\mathrm{Cos}t\;\mathrm{of}\;\mathrm{cultivation}\;\left(\mathrm{USD}\cdot {\;\mathrm{ha}}^{-1}\right)$$

B:C ratio was calculated by using the following equation:

(6) $$B:C\;\mathrm{ratio}=\frac{\mathrm{Gross}\;\mathrm{return}\;\left(\mathrm{USD}\;\cdot {\mathrm{ha}}^{-1}\right)}{\mathrm{Cos}t\;\mathrm{of}\;\mathrm{cultivation}\;\left(\mathrm{USD}\cdot {\;\mathrm{ha}}^{-1}\right)}$$

### 2.6. Statistical Analysis

The data was analysed using SPSS version 16.0 (SPSS Inc., Chicago, IL, USA) packages. Analysis of variance (ANOVA) followed by a Duncan Multiple Range test was performed to test the significant difference between the treatment results.

## 3. Results

### 3.1. Impact of Foliar Application of Zn and Fe on Grain and Straw Yield of Lentil

The two-year mean data demonstrated that the foliar application of Zn and Fe at S1 + S2 stages posed a significant impact on grain and straw yield of lentil (Table 2).

The highest value of grain yield was recorded in treatment T7 (987 kg·ha^−^^1^), which was not statistically different with the results of treatments T4 (961 kg·ha^−^^1^) and T6 (933 kg·ha^−^^1^). The minimum value of grain yield was observed in treatment T1 (688 kg·ha^−^^1^), which was not statistically different with treatment T2 (725 kg·ha^−^^1^). In case of straw yield, the highest value of two years of mean data was achieved in treatment T7 (3539 kg·ha^−^^1^), which was not statistically different from treatments T4 (3290 kg·ha^−^^1^) and T6 (3288 kg·ha^−^^1^), whereas the minimum straw yield was observed in treatment T1 (2166 kg·ha^−^^1^), which was not statistically different with treatment T2 (2417 kg·ha^−^^1^). Thus, statistically, foliar application of ZnSO_4_.7H_2_O (0.5%) + FeSO_4_.7H_2_O (0.5%) was equally effective at enhancing the grain and straw yield of lentil as FeSO_4_.7H_2_O (0.5%) at S1 + S2 stages as well as ZnSO_4_.7H_2_O (0.5%) + FeSO_4_.7H_2_O (0.5%) at S1. Also, the foliar application of ZnSO_4_.7H_2_O (0.5%) along with RDF at S1 did not significantly affect the grain and straw yield of lentil.

### 3.2. Impact of Biofortification on Grain Zn and Fe Concentration of Lentil

The two-year data for grain Zn and Fe concentration in lentil in response to foliar application of Zn and Fe at various stages of growth is given in Table 3. The results demonstrated that foliar application of Zn and Fe at S1 + S2 stages significantly enhanced the Zn and Fe concentration in lentil over the control. The results of grain Zn concentration in lentil suggested that treatment T7 (66.4 mg·kg^−1^) was most effective in comparison to treatment T1, in which the least value of grain Zn concentration (55.9 mg·kg^-1^) was observed. Thus, foliar application of ZnSO_4_.7H_2_O (0.5%) + FeSO_4_.7H_2_O (0.5%) along with RDF at S1 + S2 stages was most effective to enhance the grain Zn concentration in lentil. The results of grain Fe concentration in lentil demonstrated that the maximum Fe concentration was recorded in treatment T7 (82.9 mg·kg^−1^), which was not statistically different with treatment T4 (78.9 mg·kg^−1^) and T6 (77.6 mg·kg^−1^). The minimum Fe concentration in grain was recorded in treatment T1 (65.5 mg·kg^−^^1^), which was not statistically different from treatment T2 (68.6 mg·kg^−^^1^).

### 3.3. Impact of Biofortification on Straw Zn and Fe Concentration of Lentil

The results of two years of data concerning the Zn and Fe concentration in the straw of lentil with foliar application of Zn and Fe are given in Table 4. 

Maximum straw Zn concentration in two years was observed in treatment T7 (51.8 mg·kg^−1^), which was not statistically different with treatment T4 (47.2 mg·kg^−1^). Minimum straw Zn concentration was observed in treatment T1 (37.0 mg·kg^−1^), which was not statistically different with treatment T3 (40.0 mg·kg^−1^). Thus, foliar application of FeSO_4_.7H_2_O (0.5%) along with RDF at S1 stage did not significantly affect the straw Zn concentration of lentil. The recorded data for Fe concentration in straw showed that treatment T7 was most effective to enhance the Fe concentration in straw (159 mg·kg^−1^), which was not statistically different with treatment T6 (149 mg·kg^−1^) and T4 (148 mg·kg^−1^). The Fe concentration in straw was minimum in treatment T1 (116 mg·kg^−1^). Thus, statistically, foliar application of ZnSO_4_.7H_2_O (0.5%) + FeSO_4_.7H_2_O (0.5%) either applied at the S1 stage or S1 and S2 stages are equally effective at enhancing the Zn and Fe concentration of lentil in straw.

### 3.4. Impact of Biofortification on Grain Zn and Fe Uptake of Lentil

The Zn and Fe uptake by grain in lentil significantly increased with sole and combined use of Zn and Fe at different stages of growth as shown by the results presented in Table 5.

Maximum Zn uptake was recorded in treatment T7 (65.5 g·ha^−1^), which was not statistically different with treatment T4 (59.6 g·ha^−1^). The least Zn uptake by lentil grain was observed in treatment T1 (38.5 g·ha^−1^), which was not statistically different with treatment T2 (43.1 g·ha^−1^). The scrutiny of two-year data for grain Fe uptake in lentil demonstrated that the maximum Fe uptake was observed in treatment T7 (81.8 g·ha^−1^), and the minimum was observed in control (45.1 g·ha^−1^), which was not statistically different with treatment T2 (49.7 g·ha^−1^). Thus, foliar application of ZnSO_4_.7H_2_O (0.5%) at S1 stage did not significantly affect the grain Zn and Fe uptake of lentil. Overall, foliar application of ZnSO_4_.7H_2_O (0.5%) + FeSO_4_.7H_2_O (0.5%) either applied at the S1 stage or the S1 and S2 stages are equally effective at enhancing the grain Zn uptake in lentil.

### 3.5. Impact of Biofortification on Straw Zn and Fe Uptake of Lentil

The two-year data of Zn and Fe uptake by straw in lentil as affected by sole and combined foliar application of Zn and Fe at different growth stages has been given in Table 6. The mean data suggested that both sole Zn and combined Zn + Fe application significantly enhanced the Zn and Fe uptake in straw over the control. The maximum Zn and Fe uptake by straw was observed in treatment T7 (183.3 and 562.7 g·ha^−1^, respectively) and the minimum was observed in control (183.3 and 251.3 g·ha^−1^, respectively). Thus, foliar application of ZnSO_4_.7H_2_O (0.5%) + FeSO_4_.7H_2_O (0.5%) along with RDF at the S1 and S2 stages was most effective in enhancing the Zn and Fe uptake in the straw of lentil. Moreover, the micronutrient uptake was higher in the treatments in which FeSO_4_ (T3 and T6) was applied as compared to ZnSO_4_ (T2 and T5).

### 3.6. Impact of Biofortification on Efficiency Indices of Lentil

The results of Table 7 demonstrated that the MEI-Zn was highest in treatment T1 (1.51) and lowest in treatment T2 (1.31) which showed that the external supply of Zn has more of an effect on straw Zn concentration as compared to grain Zn concentration. The apparent recovery efficiency of Zn (ARE-Zn) was highest in treatment T2 (10.0) and lowest in treatment T3 (4.56), whereas ARE-Fe was at its maximum in treatment T3 (28.3) and its minimum in treatment T2 (12.9). The results of PE-Zn were highest for treatment T3 (18.7) and least in treatment T2 (8.20), whereas PE-Fe was at its maximum in treatment T5 (7.18) and its minimum in treatment T2 (4.77).

### 3.7. Economic Analysis

**Figure 2:**
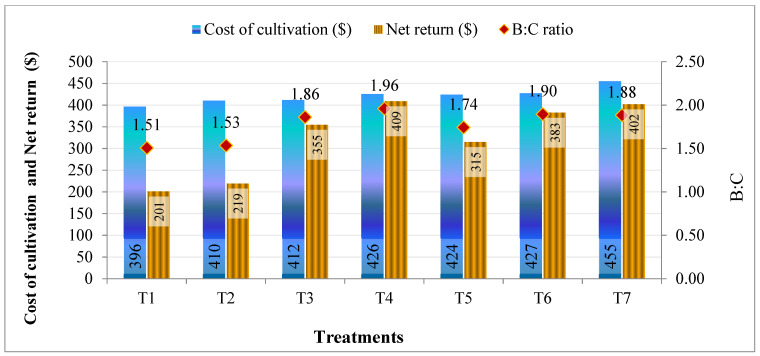
Effect of Zn and Fe biofortification on cost of cultivation, net returns and economic analysis of lentil. Treatment details are available in Table 1.

The economic analysis of lentil cultivation as affected by foliar application of ZnSO_4_ and FeSO_4_ is shown in Figure 2. The data indicated that the cultivation cost was maximum for treatment T7 ($455) and minimum in control ($396). The highest net return was recorded for treatment T4 ($409) followed by T7 ($402). The benefit:cost ratio (B:C) ratio was highest recorded in treatment T4 (1.96) followed by T7 (1.88) and least in treatment T1 (1.51)

## 4. Discussion

### 4.1. Grain and Straw Yield

Grain yield is a crucial parameter of the crop grown for commercial purposes. The results of Table 3 indicate that the combined application of ZnSO_4_.7H_2_O (0.5%) + FeSO_4_.7H_2_O (0.5%) either at S1 or at the S1 and S2 stages significantly increased the grain yield of lentils, which might be ascribed to the synergistic interactions between Zn and Fe. Zinc acts as an important structural component of various enzymes involved in the metabolism of plant growth and yield component. Zinc also plays a crucial role in sugar and protein synthesis as well as seed production. Zinc application to lentil at the reproductive phase might have induced the structural and functional alterations in pollen grains and stigma of the plants, thus increasing the seed setting of lentils in contrast to unfertilized plots [32]. Another possible reason could be the fostered photosynthetic rate and translocation of photosynthetic products to the grain with Zn application, which resulted in higher enzymatic activity and thus grain and straw yield [33]. Thus, foliar application of Zn results in its facile absorption and transportation within the plant body and improves the grain and straw yield. The higher grain and straw yield with exogenous Fe supply during the reproductive phase can be explained in association with the elevated carbohydrates and protein synthesis and their transportation towards the site of grain production. Also, the increase in Fe might have increased the activation of several enzymes, as it is a structural constituent of several enzymes involved in photosynthesis such as ferredoxin and cytochromes [34]. Apart from that, Fe encourages the seed maturation, metabolism of nucleic acid and synthesis of growth promoter molecules like auxins [33]. In good agreement with this, the increase in grain yield of several other crops has been reported in a number of previous research endeavors [23,33]. Habib recorded the highest wheat yield with a combined application of Zn and Fe as compared to the sole application of these nutrients [34]. Likewise, the combined application of 0.5% ZnSO_4_.7H_2_O and 0.5% FeSO_4_.7H_2_O was found to be more effective at increasing the grain and straw yield of chickpea as compared to the sole application of Zn and Fe. Moreover, the treatment in which fertilizers were applied at the pre-flowering and pod-formation stages was more effective as compared to the treatment when fertilizers were applied only at the pre-flowering stage [35].

### 4.2. Zn and Fe Concentration

Although the micronutrient (Zn and Fe) requirement of the crop is very small, yet it is essential for optimum plant growth. Foliar application of Zn (single or double spray) resulted in higher Zn concentrations in the grain and straw of lentil in comparison to untreated plants, which might be ascribed to the immediate absorption of Zn by plant leaves as the nutrient spray is directed toward the leaves. Higher Zn concentration in grain and straw with two Zn sprays over the single Zn spray was associated with higher Zn availability through two sprays. For instance, foliar application of micronutrients in oilseed crops at the S1 and S2 stages has resulted in a significant increase in micronutrient concentration in the edible part of the crop [36]. Another possible reason might be the suppression of the antinutrient (phytate) due to which bioavailable Zn concentration increased [31]. Similar results were observed for the Fe concentration in grain and in the straw of lentil. Higher Fe concentration in straw than grain might be associated with the presence of Fe storage proteins and non-heme proteins, which have a high binding capacity for Fe. In concordance with the present results, the combined application of Zn and Fe was found to be a superior treatment in enhancing Zn and Fe concentration in wheat as compared to the sole application of these nutrients [34]. In Indian mustard, the combined application of 0.5% ZnSO_4_.7H_2_O and 0.5% FeSO_4_.7H_2_O was found to be more effective to increase the Zn and Fe concentration as compared to the sole application of Zn and Fe. Moreover, the treatment involving the fertilizer application at pre-flowering and pod-formation stages was more effective as compared to the single spray of fertilizers at the pre-flowering stage of Indian mustard [37].

### 4.3. Zn and Fe Uptake

The results of the study showed that micronutrient (Zn and Fe) uptake has been found to increase significantly with external supplementation. The trend can be coupled with the combined effect of yield and concentration. Overall, the combined application of ZnSO_4_ + FeSO_4_ was most effective at increasing the uptake of Zn and Fe in grain and straw. Moreover, the Zn and Fe uptake was higher with foliar Fe application as compared to the foliar Zn application due to significantly higher yield with Fe supplementation.

### 4.4. Efficiency Indices and Economic Analysis

The agronomic efficiency reflects the impact of fertilizer applied on economic returns. The trend suggested that the application of 0.5% FeSO_4_ at the S1 and S2 stages is most effective at increasing the lentil production as compared to the ZnSO_4_ (0.5%) and ZnSO_4_.7H_2_O (0.5%) + FeSO_4_.7H_2_O (0.5%). The results of MEI indicated that the MEI of Zn and Fe were higher in control and the values were higher for MEI-Zn as compared to MEI-Fe, which suggests higher mobility of Zn as compared to Fe. The ARE measured the extent of nutrient loss from the cropping system and effectiveness of management practices. The results suggested that foliar application of a particular nutrient is effective in overcoming the nutrient losses. The results of PE indicate the increase in grain production with the absorbed nutrient. The higher values PE-Zn and PE-Fe were observed in treatment where the FeSO_4_ was applied as compared to the treatment in which ZnSO_4_ was applied. A similar increase in efficiency indices has also been observed in Indian mustard with Zn and Fe application [37].

The results of the economic analysis indicated that the application of ZnSO_4_.7H_2_O and FeSO_4_.7H_2_O improved the economic outcomes of lentil cultivation. The results are in alignment with previous studies, where micronutrient application has increased the B:C ratio of sesame cultivation [31]. Also, the two sprays of ZnSO_4_.7H_2_O (0.5%) + FeSO_4_.7H_2_O (0.5%) is more effective over the single spray. Moreover, the combined application of ZnSO_4_.7H_2_O (0.5%) + FeSO_4_.7H_2_O (0.5%) is more effective over the sole application of nutrients. In agreement with the present results, the combined application of 0.5% ZnSO_4_.7H_2_O and 0.5% FeSO_4_.7H_2_O was found to be more profitable as compared to the sole application of Zn and Fe for chickpea cultivation. Moreover, the double spray of 0.5% ZnSO_4_.7H_2_O + 0.5% FeSO_4_.7H_2_O at the pre-flowering and pod-formation stages was found to be more economical as compared to the single spray of 0.5% ZnSO_4_.7H_2_O and 0.5% FeSO_4_.7H_2_O at the pre-flowering stage of chickpea [30].

## 5. Conclusions

Lentil (*Lens culinaris* Medik) is a leguminous crop predominantly grown in Asia whose productivity can be influenced by the application of micronutrients in order to enhance their availability to consumers worldwide. The combined foliar application of Zn and Fe through ZnSO_4_.7H_2_O (0.5%) + FeSO_4_.7H_2_O (0.5%) at the pre-flowering + pod formation stages was found to be efficient in improving the grain and straw yield, Zn and Fe concentration and uptake, apparent recovery efficiency and benefit:cost ratio. The results of foliar application of ZnSO_4_.7H_2_O (0.5%) + FeSO_4_.7H_2_O (0.5%) at the pre-flowering + pod formation stages were statistically at par with the results obtained at the pre-flowering stage. Thus, the present study points towards the potential application of ZnSO_4_.7H_2_O (0.5%) + FeSO_4_.7H_2_O (0.5%) at the pre-flowering stages as the most effective treatment for enhancing yield, nutrient concentration and economic returns of lentil grown in sandy loam soils in northwestern India. In sum, dual-biofortified lentil can contribute significantly in increasing the bioavailability of Zn and Fe to the population at risk of Zn and Fe deficiency.

## Figures and Tables

**Table 1:** Treatment details of the experimental field at the time of sowing of lentil.

SL. No	Treatments	Stage of Application
T_1_	RDF (control)	
T_2_	RDF + ZnSO_4_.7H_2_O (0.5%) foliar spray	S1 *
T_3_	RDF + FeSO_4_.7H_2_O (0.5%) foliar spray	S1
T_4_	RDF + ZnSO_4_.7H_2_O (0.5 %) + FeSO_4_.7H_2_O (0.5 %) foliar spray	S1
T_5_	RDF + ZnSO_4_.7H_2_O (0.5%) foliar spray	S1 + S2 **
T_6_	RDF + FeSO_4_.7H_2_O (0.5%) foliar spray	S1 + S2
T_7_	RDF + ZnSO_4_.7H_2_O (0.5%) + FeSO_4_.7H_2_O (0.5%) foliar spray	S1 + S2

RDF = Recommended Dose of Fertilizers (N: 12 kg·ha^−1^, P_2_O_5_: 40 kg·ha^−1^); * Pre-flowering ** Pod formation.

**Table 2:** Effect of biofortification on grain and straw yield of lentil.

Treatments	Grain Yield (kg·ha^−1^)			Straw Yield (kg·ha^−1^)		
	Year I	Year II	Mean	Year I	Year II	Mean
T1	686 ^d^ ± 24	690 ^d^ ± 27	688 ^c^ ± 35	2139 ^d^ ± 101	2194 ^d^ ± 99	2166 ^d^ ± 94
T2	710 ^cd^ ± 37	740 ^d^ ± 31	725 ^c^ ± 38	2277 ^d^ ± 105	2560 ^c^ ± 126	2417 ^cd^ ± 103
T3	906 ^ab^ ± 43	860 ^c^ ± 42	883 ^b^ ± 41	2918 ^bc^ ±145	2780 ^bc^ ± 123	2849 ^b^ ± 138
T4	952 ^a^ ± 41	970 ^ab^ ± 48	961 ^a^ ± 47	3289 ^ab^ ± 152	3280 ^a^ ± 159	3290 ^a^ ± 161
T5	782 ^bcd^ ± 32	920 ^b^ ± 35	851 ^b^ ± 36	2459 ^cd^ ± 96	2925 ^b^ ± 134	2692 ^bc^ ± 145
T6	926 ^ab^ ± 39	940 ^ab^ ± 47	933 ^ab^ ± 43	3206 ^ab^ ± 139	3370 ^a^ ± 162	3288 ^a^ ± 138
T7	994 ^a^ ± 45	980 ^a^ ± 33	987 ^a^ ± 34	3609 ^a^ ± 163	3469 ^a^ ± 148	3539 ^a^ ± 159
LSD (*p* ≤ 0.05)	155	55	94	474	284	324

Treatments details are available in Table 1; Year I, 2019–2020; Year II, 2020–2021; The data in table represents the mean value of three replications; The values having identical superscript letter do not differ significantly at the 5% level by Duncan’s Multiple Range test.

**Table 3:** Effect of biofortification on Zn and Fe concentration in the grain of lentil.

Treatments	Grain Zn Concentration (mg·kg^−1^)			Grain Fe Concentration (mg·kg^−1^)		
	Year I	Year II	Mean	Year I	Year II	Mean
T1	50.6 ^d^ ± 2.12	60.2 ^a^ ± 3.04	55.9 ^e^ ± 2.58	64.9 ^e^ ± 3.32	66.0 ^e^ ± 3.13	65.5 ^d^ ± 3.22
T2	57.1 ^c^ ± 2.51	61.9 ^a^ ± 3.27	59.5 ^d^ ± 2.89	66.9 ^de^ ± 3.21	70.3 ^de^ ± 3.51	68.6 ^cd^ ± 3.36
T3	56.2 ^c^ ± 2.96	61.0 ^a^ ± 2.98	58.6 ^d^ ± 2.97	73.1 ^cd^ ± 3.57	74.8 ^bcd^ ± 3.89	73.9 ^bc^ ± 3.73
T4	59.5 ^ab^ ± 3.01	64.4 ^a^ ± 3.12	62.0 ^bc^ ± 3.06	81.1 ^ab^ ± 4.01	76.7 ^abc^ ± 3.38	78.9 ^ab^ ± 3.69
T5	57.8 ^bc^ ± 2.85	68.1 ^a^ ± 3.37	63.0 ^b^ ± 3.11	70.5 ^de^ ± 3.43	72.7 ^c^ ± 3.56	71.6 ^c^ ± 3.49
T6	56.4 ^c^ ± 2.49	65.6 ^a^ ± 3.01	61.0 ^cd^ ± 2.75	77.0 ^bc^ ± 3.78	78.3 ^ab^ ± 3.89	77.6 ^ab^ ± 3.83
T7	62.2 ^a^ ± 3.18	70.7 ^a^ ± 3.52	66.4 ^a^ ± 3.35	84.7 ^a^ ± 4.12	81.3 ^a^ ± 4.02	82.9 ^a^ ± 4.07
LSD (*p* ≤ 0.05)	2.9	NS	1.5	6.2	4.8	5.8

Treatment details are available in Table 1; Year I, 2019-2020; YII, 2020-2021; The data in the table represents the mean value of three replications. The values having identical superscript letter do not differ significantly at 5% level by Duncan’s Multiple Range test.

**Table 4:** Effect of biofortification on Zn and Fe concentration in straw of lentil.

Treatments	Straw Zn Concentration (mg·kg^−1^)			Straw Fe Concentration (mg·kg^−1^)		
	Year I	Year II	Mean	Year I	Year II	Mean
T1	40.4 ^d^ ± 2.52	33.6 ^d^ ± 1.87	37.0 ^d^ ± 1.05	106 ^d^ ± 4.32	126 ^e^ ± 5.96	116 ^d^ ± 5.25
T2	47.8 ^bc^ ± 2.63	43.9 ^b^ ± 2.24	45.9 ^b^ ± 2.13	119 ^c^ ± 5.03	139 ^cde^ ± 6.36	129 ^c^ ± 5.84
T3	42.4 ^d^ ± 2.04	37.5 ^cd^ ± 1.96	40.0 ^cd^ ± 1.96	138 ^b^ ± 6.34	148 ^bcd^ ± 7.19	143 ^b^ ± 6.86
T4	50.1 ^ab^ ± 2.61	44.2 ^b^ ± 2.16	47.2 ^ab^ ± 2.43	145 ^ab^ ± 6.94	150 ^bc^ ± 6.98	148 ^ab^ ± 7.52
T5	48.5 ^abc^ ± 2.45	43.2 ^b^ ± 2.12	45.9 ^b^ ± 2.07	123 ^c^ ± 5.94	135 ^de^ ± 6.78	129 ^c^ ± 6.17
T6	44.8 ^c^ ± 2.16	40.5 ^bc^ ± 2.01	42.7 ^bc^ ± 2.01	142 ^ab^ ± 7.13	155 ^ab^ ± 7.65	149 ^ab^ ± 7.33
T7	52.9 ^a^ ± 2.67	50.7 ^a^ ±2.54	51.8 ^a^ ± 2.35	152 ^a^ ± 7.37	166 ^a^ ± 7.99	159 ^a^ ± 7.21
LSD (*p* ≤ 0.05)	4.8	5.2	4.9	10	13	12

Treatments detail are available in Table 1; Year I, 2019–2020; Year II, 2020–2021; The data in the table represents the mean value of three replications; the values having identical superscript letters do not differ significantly at the 5% level by Duncan’s Multiple Range test.

**Table 5:** Effect of biofortification on Zn and Fe uptake in grain of lentil.

Treatments	Grain Zn Uptake (g·ha^−^^1^)			Grain Fe Uptake (g·ha^−^^1^)		
	Year I	Year II	Mean	Year I	Year II	Mean
T1	34.7 ^e^	41.4 ^c^	38.5 ^d^	44.5 ^e^	45.5 ^d^	45.1 ^d^
T2	40.5 ^de^	45.8 ^bc^	43.1 ^d^	47.5 ^de^	52.0 ^d^	49.7 ^d^
T3	50.9 ^bc^	52.5 ^b^	51.7 ^c^	66.2 ^c^	64.3 ^c^	65.3 ^c^
T4	56.6 ^ab^	62.5 ^a^	59.6 ^ab^	77.2 ^ab^	74.4 ^ab^	75.8 ^ab^
T5	45.2 ^cd^	62.7 ^a^	53.6 ^bc^	55.1 ^d^	66.9 ^b^	60.9 ^c^
T6	52.2 ^bc^	61.7 ^a^	56.9 ^bc^	71.3 ^bc^	73.6 ^ac^	72.4 ^b^
T7	61.8 ^a^	69.3 ^a^	65.5 ^a^	84.2 ^a^	79.7 ^a^	81.8 ^a^
LSD (*p* = 0.05)	7.4	8.4	7.6	9.6	6.7	7.0

Treatment details are available in Table 1; Year I, 2019–2020; Year II, 2020–2021; The data in the table represents the mean value of three replications; the values having identical superscript letters do not differ significantly at 5% level by Duncan’s Multiple Range test.

**Table 6:** Effect of biofortification on Zn and Fe uptake in straw of lentil.

Treatments	Straw Zn uptake (g·ha^−^^1^)			Straw Fe uptake (g·ha^−^^1^)		
	Year I	Year II	Mean	Year I	Year II	Mean
T1	86.4 ^f^	73.7 ^e^	80.2 ^e^	226.7 ^e^	276.5 ^e^	251.3 ^e^
T2	108.8 ^e^	112.4 ^cd^	110.9 ^d^	271.0 ^de^	355.8 ^d^	311.7 ^d^
T3	123.7 ^de^	104.3 ^d^	114.0 ^d^	402.7 ^c^	411.4 ^cd^	407.4 ^c^
T4	164.8 ^b^	145.0 ^b^	155.3 ^b^	476.8 ^b^	492.0 ^b^	486.8 ^b^
T5	119.3 ^e^	126.4 ^bcd^	123.6 ^cd^	302.5 ^d^	394.9 ^d^	347.3 ^d^
T6	143.6 ^cd^	136.5 ^bc^	140.4 ^bc^	455.3 ^b^	522.4 ^ab^	489.9 ^b^
T7	190.9 ^a^	175.9 ^a^	183.3 ^a^	548.6 ^a^	575.8 ^a^	562.7 ^a^
LSD (*p* ≤ 0.05)	20.2	26.5	24.5	45.5	55.6	48.8

Treatments detail are available in Table 1; Year I, 2019–2020; Year II, 2020–2021; The data in table represents the mean value of three replications; the values having identical superscript letters do not differ significantly at 5% level by Duncan’s Multiple Range test.

**Table 7:** Effect biofortification on efficiency indices of lentil.

Treatments	MEI-Zn	MEI-Fe	ARE-Zn	ARE-Fe	PE-Zn	PE-Fe
T1	1.51	0.56	-	-	-	-
T2	1.30	0.53	10.0	12.9	8.20	4.77
T3	1.47	0.52	4.56	28.3	18.7	5.62
T4	1.31	0.53	6.52	18.2	14.6	6.14
T5	1.37	0.56	6.4	7.6	11.8	7.18
T6	1.43	0.52	4.32	18.0	17.4	5.73
T7	1.28	0.52	5.06	12.1	13.0	5.69

Treatments details are available in Table 1.

## Data Availability

All data are available in the manuscripts.

## Sample Availability

Samples of the compounds (ZnSO_4_.7H_2_O and FeSO_4_.7H_2_O ) are available from the authors.
